# The assessment, serial evaluation, and subsequent sequelae of acute kidney injury (ASSESS-AKI) study: design and methods

**DOI:** 10.1186/1471-2369-11-22

**Published:** 2010-08-27

**Authors:** Alan S Go, Chirag R Parikh, T Alp Ikizler, Steven Coca, Edward D Siew, Vernon M Chinchilli, Chi-yuan Hsu, Amit X Garg, Michael Zappitelli, Kathleen D Liu, W Brian Reeves, Nasrollah Ghahramani, Prasad Devarajan, Georgia Brown Faulkner, Thida C Tan, Paul L Kimmel, Paul Eggers, John B Stokes

**Affiliations:** 1 Kaiser Permanente Northern California, Oakland, CA, USA; 2 University of California, San Francisco, San Francisco, CA, USA; 3 Yale University, New Haven, CT, USA; 4 Vanderbilt University, Nashville, TN, USA; 5 Pennsylvania State University, Hershey, PA, USA; 6 London Health Sciences Centre, Ontario, Canada; 7 Montreal Children's Hospital, Montreal, Canada; 8 Cincinnati Children's Hospital Medical Center, Cincinnati, OH, USA; 9 National Institute of Diabetes, Digestive and Kidney Diseases, Bethesda, MD, USA; 10 University of Iowa, Iowa City, IA, USA

## Abstract

**Background:**

The incidence of acute kidney injury (AKI) has been increasing over time and is associated with a high risk of short-term death. Previous studies on hospital-acquired AKI have important methodological limitations, especially their retrospective study designs and limited ability to control for potential confounding factors.

**Methods:**

The Assessment, Serial Evaluation, and Subsequent Sequelae of Acute Kidney Injury (ASSESS-AKI) Study was established to examine how a hospitalized episode of AKI independently affects the risk of chronic kidney disease development and progression, cardiovascular events, death, and other important patient-centered outcomes. This prospective study will enroll a cohort of 1100 adult participants with a broad range of AKI and matched hospitalized participants without AKI at three Clinical Research Centers, as well as 100 children undergoing cardiac surgery at three Clinical Research Centers. Participants will be followed for up to four years, and will undergo serial evaluation during the index hospitalization, at three months post-hospitalization, and at annual clinic visits, with telephone interviews occurring during the intervening six-month intervals. Biospecimens will be collected at each visit, along with information on lifestyle behaviors, quality of life and functional status, cognitive function, receipt of therapies, interim renal and cardiovascular events, electrocardiography and urinalysis.

**Conclusions:**

ASSESS-AKI will characterize the short-term and long-term natural history of AKI, evaluate the incremental utility of novel blood and urine biomarkers to refine the diagnosis and prognosis of AKI, and identify a subset of high-risk patients who could be targeted for future clinical trials to improve outcomes after AKI.

## Background

As currently defined, acute kidney injury (AKI) refers to a sudden decrease in kidney function as measured by changes in serum creatinine concentration and/or urine output. This phenomenon has been best studied among hospitalized patients and is associated with a high risk (>30%) of short-term mortality in severe cases [1]. The importance of AKI as a clinical and public health problem is underscored by recent studies showing a rising incidence in the U.S. over the past several decades [2,3].

The vast majority of published studies on AKI have focused primarily on clinical outcomes that occur during the index hospitalization complicated by AKI [4-8]. In 2005, the American Society of Nephrology Renal Research Report listed as a "critically important knowledge gap" studies addressing "long-term outcomes" after an episode of AKI [1]. Recently, several studies have attempted to examine the impact of hospital-acquired AKI on longer-term outcomes, including the risk of development and acceleration of chronic kidney disease (CKD), end-stage renal disease (ESRD), and death [9-14]. However, many existing studies have important methodological challenges that hamper their ability to determine the independent contribution of AKI to these outcomes. These include retrospective study designs, suboptimal quantification of severity of baseline CKD and severity of AKI, ascertainment of clinical outcomes based on data collected as part of routine clinical care and limited ascertainment of important confounding variables. Consequently, there has been a growing appreciation of the need to more rigorously address this question.

In 2007, the National Institute of Diabetes, Digestive and Kidney Diseases (NIDDK) released an Request For Applications (DK-07-009 "Ancillary Studies in the Natural History of Acute Kidney Injury") to create a multi-center research consortium to address these knowledge gaps by expanding our understanding of the natural history of patients suffering from AKI. The Assessment, Serial Evaluation and Subsequent Sequelae of Acute Kidney Injury (ASSSESS-AKI) Consortium was established in 2008 to compare prospectively differences in the occurrence of renal and cardiovascular outcomes and death within a diverse, matched cohort of patients with and without AKI. The overall goals of ASSESS-AKI are to:

• Establish a diverse prospective, parallel, matched cohort of adults and children with and without AKI.

• Characterize the short-term and long-term natural history of AKI based on current serum creatinine-based diagnostic criteria.

• Evaluate the incremental utility of novel blood and urine biomarkers to refine the diagnosis and prognosis of AKI.

• Develop a prognostic risk model that integrates patient characteristics and biomarkers to help inform providers and patients about the risks of adverse events after an episode of AKI.

• Identify the subset of high-risk patients who could be targeted for future interventional clinical trials to improve outcomes after an episode of AKI.

The ASSESS-AKI Study will leverage ongoing studies involving patients undergoing cardiac surgery or in the intensive care unit and expand the study population to include a more general hospitalized population to provide a broadly representative study of the natural history of AKI.

## Methods

### Study organization

**Figure 1:**
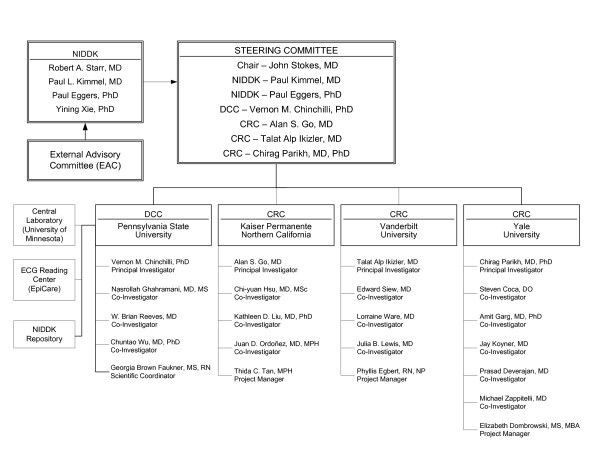
**Organizational structure for the ASSESS-AKI Study**. NIDDK = National Institute of Diabetes, Digestive and Kidney Diseases; DCC = Data Coordinating Center; CRC = Clinical Research Center network; and ECG = electrocardiography

The ASSESS-AKI Study consists of a Data Coordinating Center (Pennsylvania State University), three Clinical Research Center networks through Kaiser Permanente Northern California (Oakland, CA; San Francisco, CA; Walnut Creek, CA), Vanderbilt University (Nashville, TN), and the Translational Research Investigating Biomarker Endpoints in Acute Kidney Injury (TRIBE-AKI) network (New Haven, CT; Cincinnati, OH; London, Ontario; Montreal, Quebec). A central laboratory for analysis of core biochemistries is located at the University of Minnesota and an electrocardiography reading center is based at Wake Forest University (Figure 1). In addition, ASSESS-AKI includes an External Advisory Committee and NIDDK project scientists.

The Institutional Review Boards of the Data Coordinating Center and participating Clinical Research Centers' institutions approved the study.

### Study design

ASSESS-AKI will employ a parallel, matched, prospective cohort design of adult participants with and without AKI. In addition, ASSESS-AKI will attempt to enroll and prospectively follow all eligible children undergoing cardiac surgery requiring cardiopulmonary bypass who are participants in the TRIBE-AKI Consortium study. The study will enroll 1200 participants (1100 adults, 100 children) with approximately 50% of adult participants having AKI and the remaining 50% representing matched adult participants without AKI. Scheduled follow-up visits for each participant will occur during the subsequent four years. Informed consent will be obtained in all patients in accordance with the principles of the Declaration of Helsinki.

### Clinical research center networks

Participating Clinical Research Center Networks have active research programs related to AKI and the consortium is leveraging their existing research expertise, resources and infrastructure to establish the prospective, multi-center ASSESS-AKI cohort.

#### Kaiser Permanente Northern California

Kaiser Permanente of Northern California in collaboration with the University of California, San Francisco has been conducting a series of NIDDK-sponsored (U01DK060902, R01DK067126, R01DK058411) longitudinal studies characterizing the epidemiology and outcomes of acute, chronic, and end-stage renal disease within Kaiser Permanente's large and diverse community-based population in the San Francisco and greater Bay area. Kaiser Permanente is one of the largest integrated health care delivery systems in the U.S and provides comprehensive care for >3.2 million members that are ethnically and socioeconomically diverse and highly representative of the northern California and statewide population. Kaiser delivers comprehensive inpatient and outpatient care to its members through 18 hospitals and >60 additional ambulatory medical offices and captures many aspects of its care through the use of its comprehensive clinical (e.g., inpatient and outpatient laboratory tests) and administrative (e.g., diagnoses, procedures, mortality) databases, which will be leveraged for ASSESS-AKI. The Kaiser Permanente Division of Research will lead recruitment and enrollment of participants hospitalized in medical and surgical wards as well as intensive care units (ICU) at three Kaiser Permanente Medical Centers located in Oakland, San Francisco and Walnut Creek.

#### Vanderbilt University

Vanderbilt University Medical Center (VUMC) is a large tertiary referral center serving the middle Tennessee area and surrounding region and provides comprehensive acute and critical care services. ASSESS-AKI investigators are primarily leveraging the ongoing National Heart, Lung and Blood Institute (NHLBI)-funded (U01HL081332)-sponsored Validation of Acute Lung Injury Biomarkers for Diagnosis (VALID) Study. VALID is a single-center, prospective study whose purpose is to develop and validate a panel of diagnostic and prognostic plasma and/or urine biomarkers in a diverse cohort of 2550 critically ill patients at high risk for developing ALI/ARDS as well as AKI [15]. All adult (≥18 years) patients admitted to one of four ICUs at VUMC who remained in the ICU at day 2 were eligible for enrollment. Patients are excluded if they experienced a cardiac arrest before enrollment, had transfer orders written or anticipated within 4 hours, died or were discharged within 48 hours of ICU admission, were admitted for uncomplicated overdose, were in the ICU for >3 days before enrollment, or who had chronic lung disease requiring oxygen supplementation or pulmonary fibrosis. Per parent study protocol, blood and urine samples are currently collected at study enrollment on ICU day 2 and subsequent sampling on ICU day 4. In addition to VALID subjects, VUMC investigators are recruiting additional subjects from the same VALID ICUs as well as neurologic and burn ICUs who meet inclusion criteria for VALID.

#### TRIBE-AKI

TRIBE-AKI is an ongoing prospective cohort study of more than 1800 adults and children sponsored by the NHLBI (R01HL085757) whose goal is to validate selected biomarkers for the diagnosis and risk stratification of AKI after cardiac surgery (coronary artery bypass and/or valvular repair or replacement). Patients are excluded if they had any of the following: pre-operative AKI (≥0.5 mg/dL increase in serum creatinine concentration from preadmission to initiation of cardiac surgery); pre-operative end-stage renal disease (serum creatinine level ≥4.5 mg/dL [400 μmol/L] receiving chronic dialysis or prior renal transplant), prior cardiac transplant or insertion of left ventricular assist device; receipt of nephrotoxic agents within 48 hours preceding cardiac surgery, or acute infective endocarditis. Pediatric participants are limited to those requiring cardiopulmonary bypass. TRIBE-AKI participants have comprehensive clinical data, blood and urine samples collected pre-operatively as well as from the first five post-operative days. ASSESS-AKI will include a subset of TRIBE-AKI sites: Yale University, London Health Sciences Center (Ontario), University of Cincinnati Children's Hospital, and Montreal Children's Hospital (Quebec).

### Cohort participants

The ASSESS-AKI Study will enroll a diverse group of adults (age 18 to 89 years) and children (age one month to 18 years) with and without AKI from participating Clinical Research Center networks.

#### Baseline kidney function

To participate, all patients must have an available baseline pre-admission serum creatinine value, which is then used to estimate glomerular filtration rate (eGFR). A baseline serum creatinine value is defined as the outpatient, non-emergency department test result nearest to the index hospitalization. For the Kaiser Permanente and VALID Clinical Research Centers, the nearest value between 7 and 365 days before admission will be used, while for the TRIBE-AKI Clinical Research Center, the baseline serum creatinine can be present between 1 and 365 days before surgery, provided the patient is undergoing elective surgery. The rationale for this approach is based on preliminary data from the three Clinical Research Center networks demonstrating that the vast majority of potentially eligible participants do not have more than two to three pre-admission serum creatinine values during this time frame and that the most recent value is more likely to reflect the subject's "baseline" kidney function before the index hospitalization. All serum creatinine results must be performed using an isotope dilution mass spectrometry (IDMS)-traceable serum creatinine assay.

#### Exclusion criteria

Exclusion criteria were selected to balance the goal of maximizing representativeness of the cohort, clinical accuracy of AKI, and feasibility in achieving the project goals. These criteria are detailed in Table 1. The data sources used to ascertain information on these criteria include electronic and paper medical records, other electronic databases, and patient interviews.

**Table 1:** Inclusion and exclusion criteria for the ASSESS-AKI Study.

Inclusion criteria	Exclusion Criteria
Adult participants aged 18 years to 89 years.	Inability to provide informed or surrogate consent.
Children aged greater than one month to less than 18 years old at cohort entry.	Died prior to the three-month study visit.
Documented "baseline" serum creatinine defined as the outpatient, non-emergency department test value nearest to the index hospitalization within 7 and 365 days prior to admission using an IDMS-traceable serum creatinine assay.	Incarcerated, institutionalized, or otherwise unable to participate in the study within a home, community, or clinical setting.
In the TRIBE-AKI consortium, pre-operative serum creatinine results from an IDMS-traceable assay obtained within seven days before cardiac surgery can be used to define "baseline" kidney function for the subset of participants who are undergoing non-urgent cardiac surgery.	Enrolled in an active interventional study at the three-month in-person study visit.
For pediatric participants in TRIBE-AKI, a pre-operative serum creatinine concentration measured in the local hospital clinical laboratory among patients scheduled for elective cardiac surgery.	Actively pregnant or breastfeeding. Prior chronic hemodialysis, peritoneal dialysis (lasting ≥three months), or estimated GFR <15 ml/min/1.73 m2 not receiving renal replacement therapy. History of solid organ and/or hematopoietic cell transplants.
	Acute glomerulonephritis diagnosed clinically or by biopsy.
	Clinically significant urinary tract obstruction, confirmed by imaging.
	Hospitalization involving acute nephrectomy.
	History of multiple myeloma.
	Hepatorenal syndrome.
	Metastatic cancer or systemic cancer receiving active treatment.
	New York Heart Association Class IV heart failure prior to index admission.
	Predicted survival of 12 months or less as determined by the participant's treating physician or Clinical Research.
	Center Principal Investigator.
	AKI participants who remain hospitalized 90 or more days after the AKI episode.
	ESRD at the time of the three-month study visit.
	Unable to provide at least 1.5 mL of plasma for adults at the Inpatient visit.
	Unable to provide at least 3 mL of urine for adults at the Inpatient visit.
	Unable to provide at least 10 mL of blood for adults and 1 mL of blood for children at the three-month visit.

### Definition of AKI

We recognize the limitations of the most recently proposed definitions for AKI (i.e., RIFLE[16] and Acute Kidney Injury Network [AKIN][17]), which are based only on changes in serum creatinine concentration and/or urine output. However, despite enthusiasm for potentially more sensitive and specific novel serum and urine biomarkers,[18] to date, none have been sufficiently validated as better measures of AKI or of subsequent prognosis than serum creatinine-based AKI criteria. A major goal of ASSESS-AKI is to provide key insights into the prognostic value of novel AKI biomarkers. Therefore, AKI will be operationalized as follows which is anticipated to capture a broad spectrum of kidney injury. For adult participants, AKI will be defined as ≥50% relative increase and/or absolute increase ≥0.3 mg/dL (26 μmol/L) in peak inpatient serum creatinine compared with baseline outpatient serum creatinine. For pediatric participants, AKI will be defined as ≥50% relative increase in peak inpatient serum creatinine compared with baseline serum creatinine. We did not incorporate urine output criteria from the AKIN classification scheme because of concern about the systematic availability and quality of data about urine output, especially in non-ICU patients who are unlikely to have indwelling urinary catheters. Furthermore, incorporating the urine output criteria for AKIN might overly enrich our cohort for patients with pre-renal azotemia.

To enhance the likelihood of enrolling an adequate number of adult participants with more severe AKI, we have set an enrollment target of at least one third of AKI participants having ≥100% relative increase in serum creatinine. To increase the probability of having an adequate number of adult participants with AKI due to causes other than rapidly reversible pre-renal azotemia, we have set an enrollment target of at least one third of AKI participants who meet AKI criteria lasting ≥48 hours. These additional enrollment targets are not mutually exclusive, and we anticipate significant overlap in these pre-specified subgroups. Furthermore, study nephrologists at each participating site will review selected index hospitalization information to classify each enrolled AKI case into one of the following presumptive categories: acute tubular necrosis (ATN), pre-renal azotemia, and other/unknown.

### Definition of absence of AKI

Subjects will be considered not to have AKI if they meet the following criteria. For adult participants, non-AKI status will be defined as having both <20% relative increase and an absolute increase ≤0.2 mg/dL (18 μmol/L) in peak inpatient serum creatinine compared with baseline outpatient serum creatinine. For pediatric participants, non-AKI status will be defined as <50% relative increase in peak inpatient serum creatinine compared with baseline serum creatinine.

### Initial screening and enrollment of participants

Subject recruitment will vary by participating site and among adult versus pediatric participants, in accordance with requirements of local institutional review boards' guidelines and the requirements of sites within each Clinical Research Center network. As described above, all pediatric patients undergoing cardiac surgery requiring cardiopulmonary bypass at the two pediatric TRIBE-AKI sites will be screened for enrollment into ASSESS-AKI. Enrollment of ASSESS-AKI pediatric participants will occur post-operatively during the index hospitalization. During this first inpatient visit of enrolled children, baseline clinical data and quality of life questionnaires will be administered and 1 blood and urine specimen obtained within the first four post-operative days will be stored for future biomarker testing.

**Figure 2:**
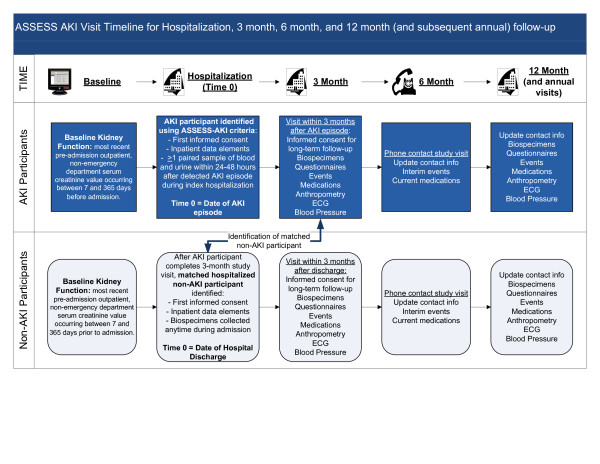
**Summary of identification and enrollment approach for AKI and Non-AKI participants**. The figure applies to adult participants only. Any eligible pediatric subject undergoing cardiopulmonary bypass-requiring cardiac surgery is approached for participation during the inpatient phase.

Among adult participants, we will enroll a parallel matched cohort of patients with and without AKI (Figure 2). Adult patients with AKI will be identified during the index hospitalization and screened for initial eligibility. During this inpatient visit, enrolled AKI patients will undergo urinalysis with microscopy through the hospital clinical laboratory and provide at least one sample of blood and urine for future biomarker testing within 96 hours of the episode of AKI.

Given the matched parallel cohort design, we will identify and enroll a sample of hospitalized adult patients who did not appear to suffer an AKI episode and who are matched in a minimum 1:1 AKI:non-AKI ratio, with each non-AKI subject individually matched to their corresponding AKI subject on the following set of key confounding characteristics: Clinical Research Center and presence of baseline chronic kidney disease using an CKD-EPI[19] equation-estimated GFR threshold of <60 ml/min/1.73 m^2^. In addition, we will further attempt to match on the presence or absence of clinical cardiovascular disease, presence or absence of diabetes mellitus, category of baseline eGFR (15-29, 30-44, 45-59, 60-89, 90-150 ml/min/1.73 m^2^), adult age category (18-39, 40-49, 50-59, 60-69, 70-79, 80-89 years), and hospital location where AKI episode occurred (ICU versus non-ICU).

### Follow-up visits and retention strategies

The follow-up visit and contact schedule are summarized in Table 2. All recruited participants will be invited to an in-person baseline study visit at 3 months after the AKI episode for AKI participants or 3 months after hospital discharge for non-AKI participants. Participants will return annually for in-person follow-up visits. Participants will be contacted by telephone at the 6-month intervals between clinic visits to obtain information on study events, or updates on general health and contact information.

**Table 2:** Summary of visit schedule and study components for the ASSESS-AKI Study

Schedule	Inpatient	Baseline	Year 1		Year 2		Year 3		Year 4	
Months	0	3	6	12	18	24	30	36	42	48
Type of Contact	Visit	Visit	Phone	Visit	Phone	Visit	Phone	Visit	Phone	Visit
Consent for inpatient visit and specimen collection	●									
Consent for long-term follow-up		●								
Update contact info	●	●	●	●	●	●	●	●	●	●
Inpatient medications	●									
Inpatient risk factors for AKI	●									
Outcome Events		●	●	●	●	●	●	●	●	●
Outpatient medications		●	●	●	●	●	●	●	●	●
Urinalysis with microscopy (adults)	●									
Urine dipstick (adults)		●		●		●		●		●
Height and weight		●		●		●		●		●
Blood pressure		●		●		●		●		●
Questionnaires										
Sociodemographic characteristics	●	●								
Lifestyle habits		●		●		●		●		●
Medical History		●		●		●		●		●
Quality of life and functional status		●		●		●		●		●
Cognitive function (adults)		●		●				●		
Electrocardiogram (adults)		●		●		●		●		●
Collect specimens for storage in biorepository	●	●		●		●		●		●
Random urine sample	●	●		●		●		●		●
Blood for DNA		●								
Serum		●		●		●		●		●
Plasma-EDTA	●	●		●		●		●		●
Plasma-citrate		●		●		●		●		●

Consistent with other cohort studies (e.g., Atherosclerosis Risk in Communities Study,[20] Cardiovascular Health Study[21]), we project that approximately 3 to 5% of participants may be lost to follow-up annually. Multiple approaches will be used to prevent participant dropout. The National Death Index will be searched periodically for all participants lost-to-follow-up to ensure complete vital status information. We will implement previously tested retention strategies to promote a high level of long-term participation. These will include free medical testing, semi-annual contact with participants via telephone calls, along with newsletters containing study updates and information about kidney disease, personalized mailings, and reimbursement of time and travel expenses.

### Collection of study data

At the outpatient visit at 3 months following the index hospitalization, adult participants will be screened again for eligibility and eligible persons will have consent obtained for long-term follow-up (Table 2). Pediatric participants will have had consent for long-term follow-up visits obtained during the index hospitalization. Information will be collected on detailed sociodemographic information, lifestyle habits, medical and family history, quality of life, current medication use, quality of life and functional status (SF-12v2™ Health Survey[22] in adults, PedsQL Generic and Cardiac modules in children),[23] cognitive function (Modified Mini-Mental Status Examination,[3MS][24] and Trails B[25]), anthropometric measures (weight, height), and resting blood pressure and heart rate. In addition, blood specimens for DNA, sera and plasma as well as a random urine sample will be obtained for local urine dipstick testing (CLINITEK Status^® ^Analyzer, Siemens, New York, NY). Sera, plasma and urine samples will be collected annually and stored for subsequent measurement of renal and cardiovascular-related biomarkers related to multiple pathways involving early AKI. The list of all the different biological specimen types that are being collected at each visit and will be stored in the NIDDK biorepository are given in Table 2.

### Biomarkers

A major goal of ASSESS-AKI is to evaluate the utility of urine and blood biomarkers for improving the diagnosis and risk stratification after a hospitalized episode of AKI. Given rapid and ongoing advances in the discovery of putative novel biomarkers for AKI,[18] the biomarkers to be evaluated within ASSESS-AKI will be prioritized based on the currently available evidence at the time of testing. The initial preliminary set of biomarkers includes those with the strongest clinical evidence base as markers of early AKI and will be measured in all study participants. Given the current data supporting the use of these markers for the detection of AKI, it will be important to know whether these markers predict short or long term outcomes. This set will include urine biomarkers (IL-18,[26] NGAL,[27] KIM-1,[28] cystatin C,[29] L-FABP[30] and NAG[31]) and blood biomarkers (serum cystatin C,[32] serum NGAL[33] and plasma IL-6[34]).

### Renal outcomes

#### Kidney function measurement

The primary renal outcome is the change in kidney function during follow-up. Kidney function will be defined before and after an AKI episode (as well as among those without AKI) using outpatient serum creatinine concentration measurements. Given the known limitations of using serum creatinine alone as a measure of kidney function,[35] except for its use in defining an episode of AKI per the criteria described previously, we will use the CKD-EPI equation to estimate GFR using an IDMS-traceable serum creatinine assay among all adult study participants. For pediatric participants, we will estimate GFR using the recommended Schwartz formula[36] based on serum creatinine values measured in local laboratories using the same assays for baseline and follow-up measurements within those sites. Urine albuminuria will be measured using a spot albumin-to-creatinine ratio [37].

#### Incident CKD

Among participants without pre-existing CKD at the index hospitalization, we will examine time to development of incident CKD with significant loss of renal function defined as experiencing both a minimum 25% reduction in level of eGFR compared with baseline and achieving CKD Stage 3 or worse[37] during follow-up.

#### Progression of CKD

Among participants with pre-existing CKD at the index hospitalization (defined as an eGFR <60 ml/min/1.73 m^2^), we will examine time to progression of CKD, defined as experiencing at least a 50% reduction in level of eGFR compared with baseline or progressing to CKD Stage 5 [37].

#### Development of ESRD

Development of ESRD after the 3 month follow-up visit will be defined as any of the following: (1) peritoneal dialysis or hemodialysis treatment at least once a week for at least 12 consecutive weeks, (2) receipt of a kidney transplant and/or (3) death while receiving dialysis.

#### Incident or recurrent episodes of AKI

We will attempt to ascertain incident and recurrent episodes of AKI. Based on available data collected during follow-up, we will use the same criteria described previously to define incident (among non-AKI participants) or recurrent (among AKI participants) episodes of AKI. However, we recognize that some study participants may be hospitalized at non-Clinical Research Center network facilities where complete medical and laboratory records may not be readily available (and where non-IDMS serum creatinine assays may be used), hindering accurate determination of whether observed changes in serum creatinine reflect progression of kidney dysfunction or a new episode of AKI. In such cases, we will pursue whether the hospitalizations included administrative diagnostic codes for AKI and acute dialysis treatments which are typically coded administratively and likely reflect more severe AKI episodes.

### Cardiovascular outcomes

To maximize future collaborations with other studies focused on kidney disease among adult populations, we have modelled our definitions after those used in the CRIC Study[38] and various longitudinal studies (Cardiovascular Health Study,[21] Atherosclerosis Risk in Communities[20] and Antihypertensive and Lipid Lowering Treatment to Prevent Heart Attack Trial[39]). The general approach will be to obtain self-reported and/or site-specific database information on potential outcome events and subsequently obtain information on qualifying *International Classification of Diseases, Ninth and Tenth Editions *(ICD-9 and ICD-10) codes at each site which will facilitate review of relevant medical records to adjudicate. The following outcomes are only relevant for the subgroup of adult participants given that they are extremely rare among children.

#### Coronary heart disease

Standard definitions will be used to classify a coronary heart disease event. This includes acute coronary syndromes such as unstable angina, non-ST-elevation myocardial infarction and ST-elevation myocardial infarction [40,41]. Myocardial infarction will be further classified according to recent international consensus guideline [42]. Sudden cardiac death will be obtained by mortality files and subject proxy contacts. It will be defined as either an unwitnessed death without another obvious cause or death occurring within one hour of the onset of ischemic symptoms per a proxy [43]. Silent myocardial infarction will be defined as new, pathologic Q waves on serial electrocardiograms (ECG)[44] among the subgroup of enrolled adult participants with the event date assigned as the mid-point between the relevant annual visits. Coronary artery revascularization will include either percutaneous coronary intervention with or without intracoronary stenting or coronary artery bypass surgery of one or more coronary blood vessels.

#### Heart failure

Heart failure will be based on hospitalizations for a clinical heart failure syndrome using relevant discharge diagnosis codes and confirmed based on Framingham Heart Study clinical criteria ascertained from medical records [45]. We will not require evidence of systolic dysfunction (e.g., left ventricular ejection fraction <40%) or diastolic dysfunction on echocardiography [46].

#### Cardiac Arrhythmias and Electrocardiographic Abnormalities

Arrhythmias and other ECG abnormalities will be based on serial ECGs using Minnesota Code definitions,[47] which have been used in epidemiological studies and have direct clinical applicability. These include development of atrial fibrillation, atrial flutter, left and right bundle branch block, atrioventricular conduction defects, and left ventricular hypertrophy among others.

### Cerebrovascular outcomes

Pertinent cerebrovascular disease outcomes include ischemic stroke and intracranial hemorrhage, and carotid endarterectomy. Ischemic stroke will be defined as acute development of a neurological deficit fitting a vascular distribution, lasting ≥24 hours, and no other evident etiology such as intracranial hemorrhage, vasculitis, tumor, or trauma [48,49]. Intracranial hemorrhage will require validation by brain imaging or pathologic evidence, and should have a documented history consistent with a stroke syndrome, diminished consciousness, or headache [50]. Carotid endarterectomy will include both surgical endarterectomy and balloon angioplasty with or without carotid stent placement.

### Peripheral arterial disease outcomes

Outcomes will include aortic aneurysm and lower extremity arterial revascularization or amputation for refractory ischemia. Lower extremity revascularization will include both percutaneous peripheral artery angioplasty and surgical arterial bypass procedures, and lower extremity amputation will include procedures performed for refractory ischemia. Hospitalizations for thoracic or abdominal aortic aneurysm dissection, rupture or repair (using percutaneous or surgical procedures) will be included.

### Mortality

Deaths will be identified primarily through surveys of subjects or their proxy contacts and review of medical records or death certificates, if available. We will also seek to obtain information on social security number from participants to conduct probability matches with Social Security Administration vital status files[51] and National Death Index[52] among the subset set of participants who are lost to follow-up. All-cause mortality will be the preferred outcome given known significant errors in assigning etiology [53].

### Statistical approach and power

The primary outcomes for the study are time-to-event outcomes, such as time to death, a renal event or a clinical cardiovascular event. Some of these events will be known exactly on a continuum and could be right-censored. Exact dates for the occurrence of some of the events, however, may only be known to occur within a specific time interval t_k_, k = 1, 2, 3, 4, 5 (t_1 _= 0-3 months, t_2 _= 3-12 months, t_3 _= 12-24 months, t_4 _= 24-36 months, t_5 _= 36-48 months). The endpoints of these five time intervals correspond to the planned in-person study visits. Therefore, the statistical analyses in these situations will invoke continuous time-to-event models that account for right-censored and interval-censored data.

The hazard function for a continuous time-to-event outcome is of the form

$$\lambda_{\text{ijk}}(\text{t};{\text{x}}_{\text{ijk}})=\lambda_{0}(\text{t})\exp({\text{x}}_{\text{ijk}}\beta )$$

where

(a) λ_ijk_(t; x_ij_) is the hazard function at time t with covariate vector x_ijk _for the k^th ^member of the j^th ^matched set within the i^th ^site, j = 1, 2,...,n_i_, and k = 0 (non-AKI), 1 (AKI),

(b) λ_0_(t) is the baseline hazard function,

(c) β is an unknown parameter vector, and

(d) x_ijk _is a vector of regressors of interest.

The regressors that will appear in x_ijk _for the primary statistical analyses are as follows using as examples different variables: binary indicator variables at month 0 for non-AKI/AKI status, CKD status, gender, Hispanic ethnicity, cardiovascular disease status, diabetes status and sepsis status; ordinal variables at month 0 for racial group (0 = white/European, 1 = Black/African American, 3 = Asian/Pacific Islander, 4 = Native American, 5 = Other/Admixed), eGFR (0 = 15 to 29, 1 = 30 to 44, 2 = 45 to 59, 3 = 60 to 89, 4 = 90-150) and albumin-to-creatinine ratio (0 = <0.15, 1 = 0.15 to 0.5, 2 = >0.5 to 1.0, 3 = >1.0 to 3.0, and 4 = >3.0), age (0 = 1 to 17, 1 = 18 to 39, 2 = 40 to 49, 3 = 50 to 59, 4 = 60 to 69, 5 = 70 to 79, 6 = 80 to 89).

The hazard model described above, however, is not the final form of the hazard model that will be applied in this study. Instead, the hazard model needs to account for (1) the dependency between an AKI subject and a non-AKI subject within a matched pair and (2) informative censoring. The occurrence of some of the renal and cardiovascular events may not be independent of the censoring event of death. For example, individuals who are censored because of death may have been at higher risk for renal and cardiovascular events. Therefore, a bivariate hazard function for the simultaneous modeling of the renal (or cardiovascular) event and death is invoked [54,55]. Let (T_ijk_, D_ijk_) denote the continuous time of a renal or cardiovascular event and the time of death, respectively, for the k^th ^member of the j^th ^pair within the i^th ^site, j = 1, 2,...,n_i_, and k = 0 (non-AKI), 1 (AKI). The bivariate hazard model is

$$\lambda_{\text{T,ijk}}(\text{t};{\text{x}}_{\text{ijk}})=\lambda_{\text{T0}}(\text{t})\exp({\text{x}}_{\text{ijk}}\beta_{\text{Ti}}+{\text{e}}_{\text{Tij}})\;\text{and }\lambda_{\text{D,ijk}}(\text{d};{\text{x}}_{\text{ijk}})=\lambda_{\text{D0}}(\text{d})\exp({\text{x}}_{\text{ijk}}\beta_{\text{Di}}+{\text{e}}_{\text{Dij}})$$

where the [e_Tij _e_Dij_]'s are independent and identically distributed according to a bivariate normal distribution with null mean vector and positive-definite variance matrix. The covariance between e_Tij _and e_Dij _represents the magnitude of the relationship between the occurrence of the renal (or cardiovascular) event and the censoring event of death. An estimated value of the covariance near zero indicates non-informative censoring, whereas an estimated value of the covariance distant from zero indicates informative censoring.

The sample size for the study is (1) 550 adult AKI subjects and 550 matched adult non-AKI subjects and (2) 50 pediatric AKI subjects and 50 pediatric non-AKI subjects, at the 3-month visit. For a two-sided, 0.05 significance level test of the relative risk equaling 1.0, the sample size of 1,200 yields greater than 80% statistical power, while allowing for a 15% withdrawal rate, for detecting a relative risk between 1.35 (when the event rate for AKI subjects is 30%) and 1.90 (when the event rate for AKI subjects is 10%).

## Discussion

Despite the emerging clinical and public health importance of AKI, studies to date have traditionally focused on characterizing its short-term consequences. Recent attempts to extend focus on longer-term outcomes have primarily involved the retrospective study of administrative databases in different population and clinical settings [9-14]. While these studies have reported a link between AKI and increased risk for either advanced CKD,[9,14] ESRD,[10-13] and all-cause death,[9-12,56-59] there have been several important limitations. Specifically, all published studies were retrospective in nature. Many did not use observed changes in serum creatinine to define AKI or rigorously quantify baseline renal function or but rather relied on administrative diagnostic codes for defining "AKI" and determining presence or absence of baseline CKD and potential confounders. Most studies included only a relatively small number of outcomes. Importantly, none of the studies collected specimens to measure biomarkers both during the AKI admission and after hospital discharge. The ASSESS-AKI study will address many of these limitations by establishing a prospective, matched parallel cohort of persons (including children) with and without AKI based on serum creatinine-based criteria measured in a standardized fashion, serial collection of blood and urine for evaluating of diagnostic and prognostic markers in AKI, and systematic follow-up for changes in renal function and multiple clinical and patient-centered outcomes.

One of the key challenges of existing studies relates to defining "baseline" kidney function for determination whether an episode of AKI has occurred and the severity of AKI [60,61]. A recent examination of this issue revealed that the use of imputed or commonly used surrogate estimates of baseline function can result in substantial misclassification of AKI and hinder adequate study of its associated outcomes [62]. Enrollment into ASSESS-AKI requires a pre-admission serum creatinine performed using an IDMS-traceable assay using a time frame recent enough to the index hospitalization that is likely to represent steady-state renal function and will substantially reduce the misclassification of the main exposure (i.e., occurrence of AKI) and of a key confounder (pre-admission CKD status) for enrolled participants, but it also creates barriers to participant recruitment. Many hospitalized patients who experience or are at risk for AKI may not have serum creatinine values available before the AKI episode, available values may not be performed using an IDMS-traceable assay, available values may be outside of our pre-specified time window (7 to 365 days before the index hospitalization), or values obtained during the eligible time window may not actually reflect steady-state renal function. All studies of AKI are affected by this challenge given that currently available consensus definitions of AKI are based on the magnitude of change of serum creatinine concentration. ASSESS-AKI will have strong internal validity given that both participants with and without AKI will have a consistently measured baseline measure of renal function that will serve as an anchor for assessment of longitudinal changes in kidney function.

As patients who suffer from AKI also tend to systematically differ from those without AKI on relevant demographic and clinical characteristics, accurate assessment of these variables is paramount to delineating the risk attributable to AKI for poor outcomes. Toward that end, existing studies based on their retrospective design and limited data quality have been susceptible to notable residual confounding and biases that limit the ability to delineate the independent contribution of AKI, especially less severe episodes, on adverse clinical outcomes. In particular, the use of administrative codes to capture the primary exposure (AKI) and critically important modifiers such as underlying CKD can lead to misclassification bias reflected in a substantial exaggeration or underestimation of the effect observed. Most studies also did not control for the presence and severity of CKD before the AKI episode, which is problematic given that CKD and CKD severity are both potent predictors of experiencing AKI as well as CKD progression, ESRD and other adverse outcomes. In addition to using standardized measurements of serum creatinine in detailing both AKI and CKD, ASSESS-AKI will also implement a 1:1 AKI:non-AKI participant matching algorithm along with advanced analytic methods that should help to mitigate major confounding for evaluating the independent impact of AKI on targeted outcomes of interest during long-term follow-up.

Current efforts to develop and validate diagnostic biomarkers that can define and predict both AKI and CKD progression based on absolute levels rather than on changes from baseline--as is necessary when utilizing serum creatinine concentrations--may mitigate the challenges of requiring a standard definition. One of the major goals of ASSESS-AKI is to evaluate the incremental utility of blood and urine biomarkers to refine the diagnosis and prognosis of AKI above current definitions using a prospective serial biospecimen collection protocol; standardized collection, processing, storage and testing methodology; and a diverse set of clinical settings and patients. Most of the recently discovered putative biomarkers of AKI are known to demonstrate time-dependent fluctuations around the time of AKI [18]. Some biomarkers are most elevated within a few hours after a known insult such as cardiac surgery (e.g., NGAL, L-FABP), while other prominent biomarkers have a delay in their expression, with peak values occurring 24-48 hours after the time of presumed injury (e.g., IL-18, KIM-1) [18]. We anticipate that the timing of the presumed renal insult will vary across the three Clinical Research Center populations that comprise ASSESS-AKI. The TRIBE-AKI cohort will, in general, experience injury occurring during or after the time of cardiopulmonary bypass; the VALID cohort may have multiple episodes of injury in the setting of critical illness, and the Kaiser Permanente cohort will have variation in timing of injury depending on the clinical setting (e.g., medical or surgical wards, oncology or cardiology service) within a general hospitalized population. Thus, our approach is tailored to promote generalizability and pragmatism for biospecimen collection, as both blood and urine will be collected on the day of or as soon as possible after the identification of clinical AKI based on serum creatinine concentration change. Based on this approach, it remains questionable whether biomarkers of current interest will be at or near their peak concentration. However, if biomarkers of AKI are to be widely used by practicing physicians, they are most likely to be initially used when there is evidence of AKI by standard diagnostic criteria to improve the diagnosis and/or prognosis of AKI. Therefore, the results from ASSESS-AKI should be broadly generalizable to clinical practice.

In addition to the challenges mentioned above, characterizing the type of AKI, which could influence short- and long-term outcomes, has been relatively understudied in epidemiologic studies of AKI. Both AKIN and RIFLE consensus criteria do not encourage discrimination of the type of AKI (i.e., ATN, pre-renal azotemia, other), implying that the etiology and type of AKI do not affect subsequent outcomes after accounting for the magnitude of change in serum creatinine concentration and/or urine output. However, ASSESS-AKI will prospectively evaluate the contribution of type of AKI on long-term clinical outcomes -above and beyond conventional measures of AKI severity. We believe that attempting to differentiate AKI "phenotype" (i.e., ATN vs. pre-renal azotemia vs. other) is important to address this knowledge gap. We also recognize that there currently exists no "gold standard" method to distinguish between phenotypes and that the same patient could have multiple contributing etiologies for AKI. Thus, we will implement several strategies, including targeted enrollment of more severe AKI based on greater change in serum creatinine concentration and/or longer duration of serum creatinine elevation that is more likely to represent ATN; urine analysis and microscopy at the time of AKI for identification of urine sediment consistent with ATN (e.g., granular casts); and standardized adjudication of cases via consultation notes, discharge summaries, and other clinical variables. Overall, the ASSESS-AKI study will provide one of the most comprehensive efforts to characterize AKI phenotype and whether it independently alters the risk of long-term clinical outcomes.

While ASSESS-AKI has numerous strengths, it also has several limitations. The cohort will be enriched with patients suffering AKI in common at-risk settings such as surrounding cardiac surgery or within the ICU as well as in more general hospitalized settings, but we will have limited power to examine whether the clinical setting modifies the association between AKI and clinical outcomes. The inclusion of children expands the age range being evaluated but is only applicable to those requiring cardiopulmonary bypass. The relatively small number of children targeted for enrollment means we will be able to detect only large effect sizes. The multi-center cohort includes various U.S. and Canadian sites and health care delivery systems, but the results may not be generalizable to all practice settings and populations. Despite various design and analytic approaches as well as standardized data collection and quality control efforts, as an observational study, we cannot rule out the impact of residual confounding and bias. However, since a randomized comparison is not possible, our prospective cohort design and systematic follow-up will provide a rigorous assessment among eligible participants. There are ongoing plans to address some of these limitations by augmenting ASSESS-AKI with possible ancillary studies (e.g., recruitment of a larger number of pediatric patients).

## Conclusions

Improving our ability to diagnose and risk stratify AKI and understanding its natural history are pressing public health issues. The ASSESS-AKI Study is well-positioned to provide novel insights into these and other areas and will create a unique longitudinal resource for the nephrology research community.

## Competing interests

The authors declare that they have no competing interests.

## Authors' contributions

ASG participated in the design of the study and drafted the manuscript. CRP participated in the design of the study and helped to draft the manuscript. TAI participated in the design of the study. SC participated in the design of the study and helped to draft the manuscript. EDS participated in the design of the study and help to draft the manuscript. VMC participated in the design of the study and statistical analytic approach and helped to draft the manuscript. CYH participated in the design of the study. AXG participated in the design of the study. MZ participated in the design of the study and helped to draft the manuscript. KDL participated in the design of the study. WBR participated in the design of the study. NG participated in the design of the study. PD participated in the design of the study. GBF participated in the design of the study. TCT participated in the design of the study. PLK participated in the design of the study. PE participated in the design of the study. JBS participated in the design of the study. All authors read and approved the final manuscript.

## Pre-publication history

The pre-publication history for this paper can be accessed here:

http://www.biomedcentral.com/1471-2369/11/22/prepub
